# A cognitive behavioral based group intervention for children with a chronic illness and their parents: a multicentre randomized controlled trial

**DOI:** 10.1186/1471-2431-11-65

**Published:** 2011-07-14

**Authors:** Linde Scholten, Agnes M Willemen, Martha A Grootenhuis, Heleen Maurice-Stam, Carlo Schuengel, Bob F Last

**Affiliations:** 1 Emma children's hospital Academic Medical Center Amsterdam, Psychosocial department, Meibergdreef 9, 1105 AZ Amsterdam, The Netherlands; 2 VU University Amsterdam, Department of Clinical Child and Family Studies, and EMGO Institute for Health and Care Research

## Abstract

**Background:**

Coping with a chronic illness (CI) challenges children's psychosocial functioning and wellbeing. Cognitive-behavioral intervention programs that focus on teaching the active use of coping strategies may prevent children with CI from developing psychosocial problems. Involvement of parents in the intervention program may enhance the use of learned coping strategies in daily life, especially on the long-term. The primary aim of the present study is to examine the effectiveness of a cognitive behavioral based group intervention (called 'Op Koers') [1] for children with CI and of a parallel intervention for their parents. A secondary objective is to investigate why and for whom this intervention works, in order to understand the underlying mechanisms of the intervention effect.

**Methods/design:**

This study is a multicentre randomized controlled trial. Participants are children (8 to 18 years of age) with a chronic illness, and their parents, recruited from seven participating hospitals in the Netherlands. Participants are randomly allocated to two intervention groups (the child intervention group and the child intervention combined with a parent program) and a wait-list control group. Primary outcomes are child psychosocial functioning, wellbeing and child disease related coping skills. Secondary outcomes are child quality of life, child general coping skills, child self-perception, parental stress, quality of parent-child interaction, and parental perceived vulnerability. Outcomes are evaluated at baseline, after 6 weeks of treatment, and at a 6 and 12-month follow-up period. The analyses will be performed on the basis of an intention-to-treat population.

**Discussion:**

This study evaluates the effectiveness of a group intervention improving psychosocial functioning in children with CI and their parents. If proven effective, the intervention will be implemented in clinical practice. Strengths and limitations of the study design are discussed.

**Trial registration:**

Current Controlled Trials ISRCTN60919570

## Background

Improvements in pediatrics and childhood surgery result in an increase of children surviving serious diseases. Consequently the number of children living with a chronic health condition is increasing drastically. Estimated prevalence ranges to a maximum of 44% (in the Netherlands approximately 500.000 (at least 14%)) [2]. Children with CI and their families face a lifetime of medical treatment and uncertainty about the future. They often have to cope with frequent hospitalizations, painful medical procedures, pharmacological interventions, school absenteeism, and restriction of activities due to the medical treatment [3]. As a result, these children may suffer from a multitude of short and long-term cognitive, behavioral and emotional problems (e.g. rumination, attention problems, and lower self-esteem) and social maladjustment. Children with CI show more submissive behavior and tend to be more socially withdrawn, in particular when they use avoidant or passive coping strategies [4].

Therefore, intervention programs that are effective in learning active coping skills to children with CI, may help to reduce psychosocial problems in these children. Based on an overview of the published research on the efficacy of available interventions there is evidence that, in general, psychological interventions produce promising positive results [5,6]. However, little is known about the mechanisms of change of these interventions and about the medium- and long-term effects. Authors also criticize the small sample sizes, the lack of correspondence between treatment objectives and their measurement, and the lack of external validation of findings [7]. Described interventions were mostly developed for a single diagnosis, such as diabetes or asthma. However, forming homogenous groups excludes underrepresented populations, and limits the sample size, which in turn limits the possibilities to prove the effectiveness and the generalizability of the program to other groups. Also children with CI may have different diagnoses and different medical treatments, but the psychosocial challenges (e.g., frequent hospitalizations, school absenteeism, restriction of activities) are mostly the same. Therefore, there should be focus on the similarities between children with chronic illness, rather than the differences between diagnosis groups [7].

Considering the limitations of existing interventions for children with CI, a standardized group-based intervention program has been developed for heterogeneous groups of children with CI, called 'Op Koers' (in English: On Track). This intervention program comprehends the learning of active coping skills, based on a cognitive behavioral approach, with the aim to support resilience and prevent psychosocial problems. A first pilot study, with 109 children and adolescents, showed positive changes on the children's wellbeing 6 months post intervention. Patients reported significantly more relaxation and positive thinking, higher social competence, and more information seeking than before the intervention [1]. Positive findings were also repeated in a homogenous group of children with inflammatory bowel disease (IBD), compared to a small control group [8]. However, in these studies outcomes were not compared to a control group and the effect sizes for disease related coping skills were relatively small. Furthermore, the intervention did not include parents, while the effects of child directed interventions can be improved by parallel programs for parents [9]. Parental support is associated with decreased levels of distress during medical procedures [11] and increased psychosocial adjustment [10] in children with CI. However, some parents of children with CI may tend to control their children and constantly try to protect them, possibly leading to adjustment problems caused by limited autonomy development [12]. Finally, parents may avoid talking with their children about negative emotions related to the disease, such as anxiety for the course of the disease, uncertainty about future (education or work) and sadness about victimization by peers. Therefore, educating parents about the importance of parental support, by learning them to talk with their child about emotions, listen and accept the child's feelings, and support and motivate the child's autonomy is expected to enhance the effect of the child intervention, especially on the medium- and long-term. As a result, a well-balanced intervention for parents was developed, parallel to the child intervention.

To assess whether the intervention program 'Op Koers' is effective, a randomized controlled trial is required. This paper describes the rationale and the design of this study. The objective is to assess the extent to which 'Op Koers' is effective in increasing or stabilizing psychosocial wellbeing in children (8-18 years) with a chronic illness, and to examine the extent to which a newly developed parental intervention enhances the effect. Primary outcomes are child psychosocial functioning, wellbeing and child disease related coping skills. Potential mediating and moderating variables are investigated in order to identify predictors of treatment effect and to evaluate potential underlying mechanisms of change [13]. Several moderating factors, such as medical characteristics (severity of the illness, diagnoses), psychological characteristics (involvement of the participant in the program), and specific characteristics (type of hospital, organization of the program, compliance to the manual) will be explored. Type of diagnoses was not related to treatment effects in the pilot study, and will be tested as a control variable in the present study. Potential mediating factors are; child quality of life, child general coping skills, child self-perception, parental stress, quality of parent-child interaction, and parental perceived vulnerability.

## Methods/design

### Interventions

#### Child intervention 'Op Koers'

'Op Koers' is based on techniques proven to be effective in behavioral and cognitive behavioral programs for children with somatic complaints [14] and in children with behavior and/or anxiety disorders [14]. 'Op Koers' has slightly different versions for two different age groups because of differences in cognitive development and age related topics. The first group consists of primary school children (8-12 years) and the second of adolescents in secondary school (12-18 years). The intervention takes place in the hospital were the child is medically treated, and involves six weekly 90 minutes sessions, and one return session after six months. To stimulate group processes and because of educational reasons groups with a minimum of four and a maximum of eight children are formed. The intervention aims to empower children with CI by teaching the active use of coping strategies. These coping strategies are translated into five learning goals: 1) information seeking and information giving about the disease ('good to know better' principle), 2) use of relaxation during stressful situations (using exercises), 3) increase knowledge of self-management and compliance, 4) enhancement of social competence (group discussions, role playing), and 5) positive thinking (effective use of the Thinking-Feeling-Doing model; replacement of inaccurate thoughts) [14]. During the group sessions the goals are translated into psycho-education (such as informative video's and group discussions), and reinforced and practiced through exercises (such as role-play and board games) and homework assignments. In all sessions age-appropriate examples are used.

#### Parent intervention

The parent intervention is built on existing cognitive behavioral programs for parents of children with anxiety problems [15,16] and on outcomes from several parent focus groups and expert clinical advisory groups. Primary purpose of the parental module is enhancing intervention effects of the children's program, by teaching parents to be sensitive to their children's needs, and encourage their children in using the learned skills. Overall, the parent intervention is intended to change the context for the child. This is done by enhancing availability of parental support as perceived by the children, expected to result in increases in children's perceived self-esteem and in the use of active coping skills in daily life as well as in situations related to their disease and treatment, which in turn will improve social-emotional functioning. The parent intervention fits into the learning goals of the child intervention. Three learning goals are central to the parent training: 1) Learning: to understand what the children learn (psycho education, group discussions), 2) Observing: to be sensitive to children's cognitions and feelings (by assignments to talk about feelings with their child), 3) Motivating: to stimulate their children to apply the learned skills in daily life (by assignments to support their child to ask questions to the doctor). To limit the number of visits to the hospitals and to enable shared homework assignments, the parent intervention groups are organized at the same time as the intervention groups of their child, in another room.

The content of the child and parent interventions is summarized in Table 1.

**Table 1:** The five basic learning goals of 'Op Koers' (child and parent intervention) and examples of learning activities in the child and parent intervention programs.

	Examples Of learning Activities\Five Basic learning Goals	Information seeking and information giving about the illness	Use of relaxation during stressful situations	Increase knowledge of self-management and compliance	Enhancement of social competence	Positive thinking
Examples of learning activities of the child intervention	Instruction/modeling	Education about sources of information	Relaxation exercise (CD)/practicing tricks for medical procedures (video)	Discussion about own treatment and (non) Compliance	Video: take initiative and inform	Thinking-feeling-doing game
	
	Reinforcement/Practice	Role play: ask your own questions to your pediatrician	Homework: practice the relaxation exercise	Group exercise: make a drawing about your treatment	Role play: inform peers about what you cannot and what you CAN do instead	Homework: write down your negative thoughts and explore ways to get rid of them

Examples of learning activities of the parent intervention	Instruction/modeling	Education about sources of information	Relaxation exercise (CD)	Group discussion about what to do when your child has problems with compliance	Video: take initiative and inform	Introducing Thinking-feeling-doing model
	
	Reinforcement/Practice	Homework: stimulate and support your child to find and give their own answers about their illness	Homework: observe and ask your child about a stressful situation and talk about what to do	Homework: talk with your child about treatment and find out the reasons for non-compliance	Homework: practice with your child how to ask other children to play together	Talk about cognitions with your child and help to find positive thoughts

### Study design

The design is a multi centre randomized controlled trial with three conditions: 1) the child intervention group 'Op Koers', 2) the child intervention with a parallel parent program, and 3) a wait-list control group. Subjects in the control condition are placed on a one year waiting list. After the 12 months follow-up they have the opportunity to participate in the intervention. Participants assigned to the control group are not prevented to seek individual treatment, but the participating hospitals are advised to wait with individual psychological treatment until after the follow-up assessment. If the child or the family needs acute psychological care, this will be approved. It will be extensively documented and controlled in the analyses, when children and/or parents receive treatment during this period.

This study was approved by the Medical Ethics Committees of the Academic Medical Centre Amsterdam and of the participating hospitals.

### Procedure

Eligible participants are recruited from outpatient clinics from three academic hospitals and four non-academic hospitals, representing the major regions of The Netherlands. Children and parents receive an information letter from the pediatrician and posters and pamphlets are available at the clinic. Recruitment is coordinated by local investigators of each hospital. Parents and children are asked to read the information and, if interested to participate, to return an application form in a stamped self-addressed envelope. Then informed consent forms are sent to be signed by both parents and child, and within 2 weeks, a telephone interview is used to check inclusion criteria. Eligible participants are randomized to either the interventions or the wait-list control condition, and are informed about the randomization outcome by letter. In all conditions assessments take place at baseline, 6 weeks, 6 months and 12 months after baseline. Within a month after the baseline measurements, the intervention starts. Families receive a financial reward (€65) for completing assessments, spread across the four measurement points but increasing in amount at each follow-up.

Interventions are conducted by two child psychologist or by a psychologist and a psychological assistant working in the participating hospitals. The intervention is described in a manual, session by session. A protocolled training is given to all psychologists. This training consists of three parts 1) teaching the main principles of cognitive-behavioral group therapy, 2) giving more specific information on the procedures and goals related to the different sessions using video examples and the extensive manual for psychologists, 3) practicing a number of assignments to enlarge their preparation for giving the intervention. To ensure treatment integrity, randomly selected therapy sessions are recorded on audiotape or videotape, and coded afterwards following the procedure of Wood et al. [16].

**Figure 1:**
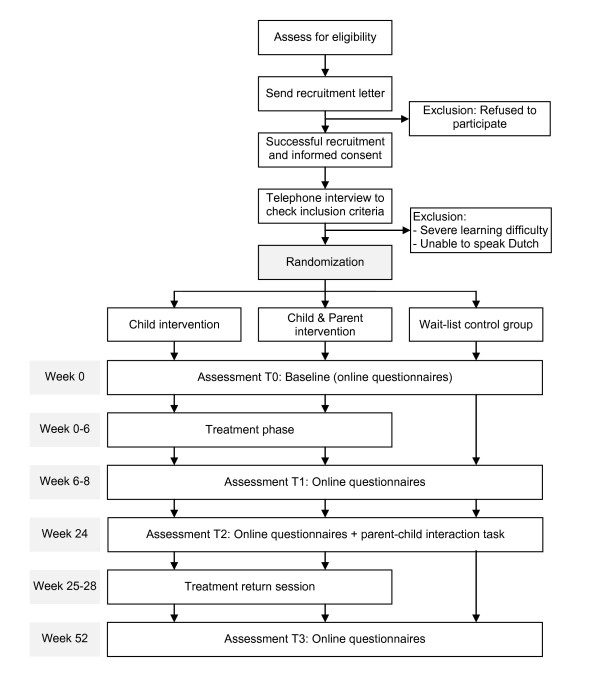
**Study procedure in flow diagram**.

Figure 1 depicts the different stages of the research procedure.

### Inclusion and exclusion criteria

For inclusion, children between 8 and 18 years old and suffering from a chronic illness are selected. The term 'chronic illness' refers to illnesses that require at least 6 months of continuous medical care, permanent life style changes and continuous behavioral adaptation to the unpredictable course of the illness [2]. Children who attend special education due to severe learning difficulties are excluded, because for these children an adapted or individual program might fit better to their individual cognitive needs. The final criterion is that children and parents should be able (with help) to fill in Dutch questionnaires.

### Randomization

Participants are randomly allocated to the conditions within their own hospital. For each hospital, the assignment of participants is carried-out by using a block randomization method [17,18]. Interventions are organized at three time points (three cohorts), to spread recruitment over time. An allocation schedule is formulated in which the conditions (separately for each age group) will equally be divided across the centers and cohorts to ensure the number of groups is about the same within each condition. To ensure minimal differences between hospitals, participants will be considered in blocks of 12, 15, 18, 21 and 24 (4 to 8 in each condition), and in each centre heterogeneity of medical diagnosis is guaranteed. An independent researcher makes the allocation schedule using random allocation software.

### Sample size

The expected effect size and attrition rates are based on former trials that studied the effects of cognitive-behavioral group interventions for children with CI. Generally, psychological interventions for children with CI indicated medium effect sizes (mean ES = 0.71, range = 0.28-3.23, SD = 0.61) [6]. In a study that evaluated cognitive-behavioral group interventions for children with anxiety disorders, enhanced by parental involvement also medium effect sizes were found [19]. Based on four repeated measurements with within-subject correlations of .5, a sample size of 126, 42 in each condition, is necessary to achieve adequate power (.80) to trace differences of medium effect size (d = .5) between the conditions over time at a significance level of .05 [20]. To be able to control for dependency effects (block and centre effects) and taking into account 10% attrition over time, 162 children have to be recruited to reach the required sample size.

### Outcomes measures

Participating children, and one of their parents, are asked to complete questionnaires at baseline, 6 weeks, 6 months and 12 months after baseline. Primary caregivers are asked to fill in questionnaires and to participate in the parent intervention. All questionnaires are assessed online; participants receive an email with a unique link to the questionnaires. Total duration time for filling in the questionnaires is estimated on 45 minutes for children and 35 minutes for the parents.

#### Questionnaires

For this study an adapted version (12 items) of the Coping Strategies Inventory (CSI) will be used [21]. All other outcome measures will be assessed by standardized questionnaires with good psychometric qualities, and available normative data [22-33] (Table 2).

**Table 2:** Primary and secondary outcome measures, measurement instruments, and informant.

Primary outcome measures	Measurements	Informant
Psychosocial functioning	Child Behaviour Checklist (CBCL) [22]	parent
	Youth Self-Report (YSR) [23]	child (age11-18)
	Strengths and Difficulties Questionnaire/4-18 (SDQ parent and child report) [24,25]	parent & child (11-18)
Disease related coping skills	Questionnaire Op Koers for children (QOK-c, 18-items) [1]	child (8-18)
	Questionnaire Op Koers for parents (QOK-p, 18-items) [1]	parent
		
**Secondary outcome measures**	**Measurements**	**Informant**

Quality of life	KIDSCREEN-27 [26]	child (8-18)
	DISABKIDS Chronic Generic Measure - short form (DCGM-12) [27,28]	child (8-18)
General coping skills	Adapted version of the Coping Strategies Inventory (CSI) [21]	child (8-18)
Self perception	Self-perception Self-Perception Profile for Children (CBSK) [29]	child (8-12)
	Self-Perception Profile for Adolescents (CBSA) [30]	child (12-18)
Parental stress	Nijmegen Parenting Stress Index-Short (NPSIS) [31]	parent
Parent - child interaction	Family Interaction Task (FIT) is a 30-minute semi-structured observation¹ [34]	observation
	Security Scale [32]	child (8-18)
Perceived vulnerability	Dutch version of the Parental Vulnerability Scale (8 items) [33]	parent

#### Parent-child interaction observation task

Six months after baseline assessment (T2) parent-child dyads are observed with a semi-structured parent-child interaction task. This task is an 30-minute observation paradigm in which parent and child collaborate on three different tasks: guiding marbles through a labyrinth, naming coping solutions for hypothetical situations using three different vignettes, and talking about personal disease related emotions and solutions. The three tasks differ in the level of collaboration and competition between parent and child. Observations of the interaction will be independently coded by trained graduate students, unaware of the child's treatment condition. In a study with 100 children (10-16 years old) with a wide range of psychological problems, this task has shown to be sufficient in identifying individual differences in parental responsiveness and granting autonomy support, child's positive affect and showing autonomy, and dyadic collaboration. Trained master students have shown to be reliable in administering and coding the observations (ICC = .74, range .66-.78) [34].

### Statistical analyses

The analyses will be performed on the basis of an intention-to-treat population. Treatment effects (group × time) will be assessed with linear mixed model analysis using SPSS. Intraclass correlations will be computed to test possible dependency effects of block randomization [35]. In secondary analyses, mediator variables will be included to investigate which underlying processes in children and families change as a result of the intervention. Moderator variables will be included to examine whether the effects are associated with characteristics of the child and the family (gender, age, medical diagnosis, severity of disease, attendance during group sessions).

## Discussion

This paper outlines the study protocol for a multicentre randomized controlled trial on the effects of a cognitive-behavioral based group intervention for children with chronic illness and their parents. Former studies have shown that psychological interventions for children and adolescents with chronic medical conditions can improve social-emotional functioning [5,6]. However, more research is needed into the effectiveness of such programs with a large sample size, adequate methodological and statistical analyses and an extended follow-up. Although it is known parental involvement enhances the use of coping strategies by the child [19] studies on interventions for parents, specifically directed at parental support and behavior, are limited [36]. Therefore the strength of the 'Op Koers' intervention is that it focuses on both the coping strategies of the child and manipulates the level of parental support. This will allow testing the assumption that involving the context bolsters long-term effects.

Another strength of the present study is that by focusing on heterogeneous groups (children with different medical diagnoses), and by including multiple centers, it is possible to include a relatively large sample size, sufficient for comparing the two intervention conditions to the control group. Also the use of a stratified block randomization method achieves to overcome practical difficulties that are common in these kinds of trials. To understand why and for whom the intervention may work, the effects of mediating and moderating factors are investigated, which makes it possible to further develop and extend the intervention program. Other strong aspects of the study are the number of assessments, and the relatively long term follow-up.

This study design has several methodological vulnerabilities. First, because of the randomization into three cohorts and the two different age groups, it may take a significant time period to recruit enough participants. This can increase the time between recruitment and actual participation and may lead to dropout attrition. Second, due to the relatively long follow-up period it is possible that participants within the control group, as well as participants in the intervention groups during the follow up period, will seek other psychosocial support. These limitations have to be taken account in data analyses.

In conclusion, children with CI are vulnerable for psychosocial problems; therefore evidence based interventions are needed. This study aims to contribute by investigating an intervention for children and their parents. If this study indeed shows significant improvement on psychosocial wellbeing and disease related coping skills, 'Op Koers' will be made available for implementation in clinical practice.

## Competing interests

The authors declare that they have no competing interests.

## Authors' contributions

All authors participated in the design of the study. LS drafted the manuscript. AMW, MAG, HMS, CS and BFL edited the manuscript. All authors read and approved the final manuscript.

## Pre-publication history

The pre-publication history for this paper can be accessed here:

http://www.biomedcentral.com/1471-2431/11/65/prepub
